# Double inhibition of XIAP and Bcl-2 axis is beneficial for retrieving sensitivity of renal cell cancer to apoptosis

**DOI:** 10.1038/sj.bjc.6604268

**Published:** 2008-02-19

**Authors:** V Bilim, K Yuuki, T Itoi, A Muto, T Kato, A Nagaoka, T Motoyama, Y Tomita

**Affiliations:** 1 Department of Urology, Yamagata University School of Medicine, Iida-nishi 2-2-2, Yamagata 990-9585, Japan; 2 Division of Molecular Oncology, Department of Signal Transduction Research, Graduate School of Medical and Dental Sciences, Niigata University, Asahimachi 1-757, Niigata 951-8510, Japan; 3 Department of Human Pathology (Second Department of Pathology), Yamagata University School of Medicine, Iida-nishi 2-2-2, Yamagata 990-9585, Japan

**Keywords:** renal cell carcinoma, apoptosis, Smac, XIAP, molecular targeting

## Abstract

Renal cell carcinoma (RCC) is known to be resistant to chemo- and radiotherapy due to a high apoptotic threshold. Smac and XIAP (X-linked inhibitor of apoptosis protein) proteins were detected in all RCC cell lines and tissue samples examined. We modulated the function of XIAP, either through its constitutional downregulation with an shRNA vector or by applying a Smac-mimicking peptide. Among RCC cell lines, Caki1 expresses the highest levels of XIAP. We transfected Caki1 with XIAP-targeting shRNA vector and generated stable clones. XIAP was knocked down by RNA interference in clone no. 14 by 81.6% and in clone no. 19 by 85.3%. Compared to the parental and mock-transfected cells, neither clone was more sensitive to conventional chemotherapeutic agents, but both clones were more susceptible to Fas stimulation (*P*<0.0001) and to pharmacological Bcl-2 inhibition (*P*<0.0001), as well as to a combination of the two (*P*<0.0001). Mature Smac binds to XIAP via the N-terminal residues, disrupting its interaction with caspases and promoting their activity. We determined that exposure of Caki1 cells to Smac-N7 peptide (AVPIAQK) resulted in a slight but significant decrease in viability (*P*=0.0031) and potentiated cisplatin's effect (*P*=0.0027). In contrast with point targeting of XIAP by shRNA, Smac-N7 peptide is active against several IAP (inhibitor of apoptosis protein) family members, which can explain its role in sensitising cells to cisplatin. Our results suggest that multiple targeting of both Bcl-2 and XIAP or, alternatively, of several IAP family members by the Smac-N7 peptide is a potent way to overcome resistance of RCC to apoptosis-triggering treatment modalities, and might be a new tool for molecular targeted therapy.

Being highly refractory to radio- and cytotoxic chemotherapy, renal cell carcinoma (RCC) poses a significant problem for conventional cancer treatment. Recently, molecular targeting therapy employing multi-kinase inhibitors (MKIs) has raised hope for patients with advanced RCC. Tyrosine kinase inhibitors such as sorafenib and sunitinib (Motzer and Bukowski, 2006; Ljungberg *et al*, 2007) are becoming first-line treatments for metastatic RCC. However, as soon as resistance to MKIs develops, the patient rapidly succumbs to the disease. It is now of utmost importance to find ways to control cancer progression. Manipulation of the apoptotic machinery is one promising approach.

Renal cell carcinoma expresses high levels of Bcl-2, which is one of the early discovered and thoroughly studied inhibitors of apoptosis. We have reported previously on the Bcl-2 status in RCC (Tomita *et al*, 1996a) and discovered that the absence of Bcl-2 favours the response of RCC to immunotherapy (Maruyama *et al*, 2006).

XIAP (X-linked inhibitor of apoptosis protein) is the most downstream inhibitor of apoptosis and is considered the most potent and ubiquitous caspase inhibitor among the members of the IAP (inhibitor of apoptosis protein) family. The translation of XIAP is stimulated under different conditions of cellular stress (Holcik *et al*, 1999) and overexpression of XIAP can be an important event in cancer progression and resistance to treatment (Holcik *et al*, 2000).

Members of the IAP family seem to be attractive targets for anticancer therapy because tumour cells rely heavily on IAP activity compared with normal cells (Yang *et al*, 2003a). XIAP (Harlin *et al*, 2001), cIAP-1 (Conze *et al*, 2005), and cIAP-2 (Conte *et al*, 2006) knockout mice are phenotypically normal. The knockout phenotype can be revealed only under specific conditions in some tissues and cells (Cummins *et al*, 2004; Conte *et al*, 2006; Zhao *et al*, 2007). Tumour cells have increased levels of spontaneous apoptosis and intrinsic caspase activity (Yang *et al*, 2003a). By inhibiting XIAP, it is possible to kill tumour cells without additional impact, as we demonstrated previously in urothelial cancer (UC) (Bilim *et al*, 2003). Moreover, XIAP antisense oligonucleotide chemosensitised UC to adriamycin. It has also been shown that XIAP expression levels increased with the progression of RCC (Ramp *et al*, 2004; Yan *et al*, 2004; Mizutani *et al*, 2007). In addition, an XIAP antisense oligonucleotide sensitised RCC to Fas/TRAIL-induced apoptosis (Mizutani *et al*, 2007). Antisense oligonucleotides directed against XIAP sensitise cancer cells to chemotherapeutic drugs *in vitro* (McManus *et al*, 2004) and those developed by Aegera Therapeutics Inc. are currently being evaluated in phase I and II clinical trials (Cummings *et al*, 2005; Schimmer *et al*, 2006).

There is a group of proteins phylogenetically conserved among insects and mammals that are natural inhibitors of IAPs and that share homology at the N terminus, which is critical to their function. This active sequence binds to the BIR domain of IAPs, disrupting interaction with both pro- and active caspases, thus promoting their activation and function. The mammalian protein Smac/DIABLO is encoded by a nuclear gene and translocated to mitochondria, where its N-terminal mitochondrial targeting sequence (MTS) is cleaved to reveal an active site (AVPIAQK). It is released from mitochondria when the cell is exposed to apoptotic stimuli and this release is antagonised by Bcl-2. Smac expression is downregulated in RCC (Mizutani *et al*, 2005). *In vitro*, peptides containing N-terminal residues of mature Smac/DIABLO have been shown to promote apoptosis by blocking IAP function.

However, there is still no strong evidence for efficacy of XIAP downregulation as a potential approach to treating urological malignancies. Here, we further analysed the functional significance of XIAP in RCC cell lines, with special emphasis on the *in vitro* targeting of XIAP by utilising recently invented technologies such as RNA interference (RNAi) and the cell-permeable oligopeptide-mimicking Smac N-terminal sequence.

## MATERIALS AND METHODS

### Immunohistochemistry

The study was approved by the Ethics Committee of Yamagata University and all patients signed an informed consent form. The surgical specimens from 34 consecutive patients who underwent surgery (15 open, 19 laparoscopic; 26 radical nephrectomies, 8 nephron-sparing surgeries) for RCC from January to December 2005 at the Yamagata University Hospital were included in the study. There were 22 males and 12 females, ranging in age from 28 to 80 (median, 62) years. Pathological staging was determined according to the UICC TNM classification of malignant tumours. Twenty-six tumours were postoperatively diagnosed as stage 1, 5 as stage 2, and 3 as stage 3a. Pathological grades were assigned according to a system developed by the Japanese Urological Association based on the degree of atypia of tumour cells. There were 29 clear cell tumours, 2 chromophobes, and 3 papillary RCCs.

Monoclonal mouse antibodies for XIAP (clone 48) and Smac/DIABLO (clone 7) (BD Transduction Laboratories, San Diego, CA, USA) were used. Two 5-*μ*m-thick paraffin sections from different parts of each tumour representative of the entire tumour were used. Epitops were reactivated by autoclaving sections in 10 mM citric buffer (pH 6.0) for 10 min. Bound primary antibody was detected by the peroxidase method using the Histofine simple stain MAX-PO (Nichirei, Tokyo, Japan). The staining reaction was developed by DAB and nuclear counterstaining was performed with haematoxylin. Positive and negative controls were included in each staining series. Adjacent normal kidney served as an internal positive control in selected sections.

The intensity of XIAP staining, established previously by Ramp *et al* (2004), was determined for both XIAP and Smac. The intensity was scored as follows: 0, no staining; 3, strong staining comparable with that of convoluted tubule epithelial cells of normal kidney; and 1 and 2, weak and moderate staining, respectively, that is, intermediate between the former two. The proportion of stained cells expressed as a percentage multiplied by the intensity index produced the staining score. Two different sections from each tumour were examined by immunohistochemistry (IHC), and the mean score for each patient was included in the statistical analysis.

### Cell culture

Established renal cell cancer cell lines ACHN, KRC/Y, Caki1, Caki2, A704, and A498 were obtained from ATCC (Rockville, MD, USA) and cultured as described previously (Tomita *et al*, 1996b). The small-molecule Bcl-2 inhibitor HA14-1 was purchased from Calbiochem (San Diego, CA, USA), CH11 was from MBL (Nagoya, Japan), cisplatin was from Nippon Kayaku (Tokyo, Japan), and docetaxel was from Sigma-Aldrich Japan (Tokyo, Japan).

### RT–PCR

Total RNA from RCC cell lines was isolated using the SV Total RNA Isolation System (Promega, Madison, WI, USA). The integrity of the extracted RNA was confirmed by gel electrophoresis. Two micrograms of total RNA was subjected to RT–PCR using a first-strand cDNA synthesis kit (Roche Molecular Biochemicals, Indianapolis, IN, USA) and AmpliTaq™ (Perkin Elmer, Branchburg, NJ, USA), as described elsewhere. To ensure appropriate first-strand cDNA synthesis, the *β*-actin gene RT–PCR was performed as described by Raff *et al* (1997). Bcl-2 family members were amplified using an ApoPrimer Set (Takara Bio, Shiga, Japan) according to the manufacturer's instructions. IAP family primers were according to Ka and Hunt (2003).

### Immunoblotting

Immunoblotting was performed as described previously (Tomita *et al*, 1996b). Horseradish peroxidase-labelled secondary antibody was detected using a SuperSignal West Pico Substrate (Pierce, Rockford, IL, USA) according to the manufacturer's instructions. *β*-Actin was used as a loading control. The images were analysed using *UN-SCAN-Itgel* Automated Digitizing System software (version 5.1 for Windows, Silk Scientific Inc., Orem, UT, USA). The following antibodies were used: anti-Bcl-2 (clone 124) from DAKO (Tokyo, Japan); polyclonal anti-c-IAP1 (no. 4952), anti-caspase 3 (no. 9662), and anti-survivin (no. 2802) from Cell Signaling Technology (Danvers, MA, USA); anti-PARP (clone 7D3-6), anti-XIAP (clone 28), anti-Smac (clone 7), anti-caspase 3 (clone 19), anti-Bcl-x (clone 44), anti-Beclin (clone 20), anti-Bad (clone 48), anti-Bax (clone 3), and anti-c-IAP2 (clone F30-2285) from BD (Franklin Lakes, NJ, USA); and anti-*β*-actin from Abcam Inc. (Cambridge, MA, USA).

### RNAi

Three XIAP-targeting short hairpin RNA vectors were generated using pcPURU6beta-i-cassete with the target sequences 5′-GTAGAAGAGTTTAATAGAT-3′ (TA0025-1), 5′-GCCGGAATCTTAATATTCG-3′ (TA0025-2), and 5′-AGGTGAAGGTGATAAAGTA-3′ (TA0025-4) by Takara Bio. Transfection was carried out using *Trans*IT-LT1® transfection reagent (Mirus Bio Corporation, Madison, WI, USA). In preliminary experiments, TA0025-4 demonstrated the highest ability to suppress XIAP and was selected for further experiments. For generation of stable transfectant clones, the transfected cells were selected with puromycin for 3–4 weeks. Twenty selected clones were screened for XIAP expression, and clone nos. 14 and 19 with the lowest levels of XIAP (20.6 and 14% of the control level, respectively) were selected for further experiments. We also isolated puromycin-resistant mock transfectants, produced by transfection of the vector carrying irrelevant sequence (5′-CACCTTTTTTT-3′) with no mammalian homology.

### Peptide transfection

The N-terminal residues of mature Smac/DIABLO are critical to its function. Smac-N7 peptide (AVPIAQK) and a functionally inactive control peptide (MVPIAQK) were synthesised by Sigma-Aldrich Japan. To deliver the peptides to cultured cells, the following two transfection reagents were used: Chariot (Active Motif, Tokyo, Japan) and BioPORTER (Sigma-Aldrich). The cell-permeable peptide Smac-N7 (AVPIAQK-PRQIKIWFQNRRMKWKK) bound to an antenopedia sequence facilitating incorporation into the cells was from Calbiochem.

### Measurement of cell viability and cell proliferation

Cell viability was detected with a colorimetric assay, the CellTiter 96® AQueous One Solution Cell Proliferation Assay (Promega), utilising tetrazolium compound according to the manufacturer's instructions. Each dose was examined in four or six wells of a 96-well plate (BD Falcon, Franklin Lakes, NJ, USA). Each experiment was repeated at least three times and representative results are presented. For estimation of cell proliferation, BrdU cell proliferation assay (Calbiochem) was carried out according to the manufacturer's instructions. The experiment was repeated twice in quadruplicate.

### Detection of apoptosis

Cells were cultured in Lab-Tek Chambers (Nunc Inc., Naperville, IL, USA) and treated with the peptide or cisplatin as described above. Apoptotic morphological changes were detected with Giemsa (Sigma-Aldrich) (3% in 15 mM phosphate buffer pH 6.8) and Hoechst 33342 (Dojindo Laboratories, Kumamoto, Japan) staining followed by observation under light and fluorescence microscopes, respectively. For the detection of early apoptosis, cells were stained with the Annexin V FITC Apoptosis Detection Kit I (BD) according to the manufacturer's instructions. Propidium iodide (PI) staining of the fixed cells, as described elsewhere, was carried out for quantification of late apoptotic events. Stained cells were analysed on FACSCalibur™ Flow Cytometer (BD).

### Statistical analysis

Continuous variables are presented as mean±s.d. Except for IHC scores, which were considered nonparametric and analysed using the Kruskal–Wallis test, all other continuous variables in the present study met the criteria for a normal distribution and were assumed to be parametric. They were analysed using a two-tailed *t*-test or one-way ANOVA where appropriate. In cases where the groups were ordered and equally spaced (as the data presented in Figure 6), the post-test for a linear trend was performed; otherwise, a Tukey post-test to compare all pairs of values was used (as the data presented in Figure 5A and B). *P*-values lower than 0.05 were considered statistically significant. Analysis was performed using GraphPad Prism version 3.02 for Windows (GraphPad Software Inc., San Diego, CA, USA).

## RESULTS

### XIAP and Smac expression in tissue samples and cell lines

**Figure 1:**
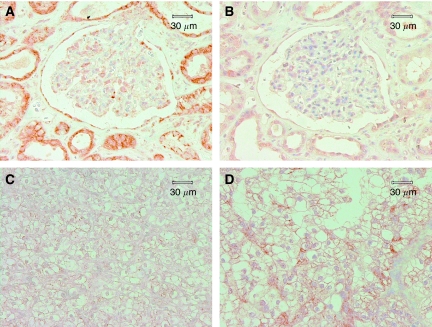
Immunohistochemical examination of Smac (**A**, **C**) and XIAP (**B**, **D**) in normal kidney (**A**, **B**) and RCC (**C**, **D**). In normal kidney, Smac expression was detected in tubular epithelial cells, Bowman capsule cells, and a portion of glomerular cells (**A**). In tumours, Smac staining was weaker than in normal kidney (**C**). XIAP expression was restricted to tubular epithelial and several glomerular cells in normal kidney (**B**).

By IHC, XIAP expression was found to be restricted to tubular epithelial and several glomerular cells, and Smac expression was detected in tubular epithelial cells, Bowman capsule cells, a portion of glomerular cells, and fibroblasts in normal kidney (Figure 1). In tumourous tissues, Smac was found in all tumours, with staining scores ranging from 200 to 300 and staining intensity weaker than in normal kidneys. Smac expression did not vary between different tumour stages and grades. XIAP expression levels increased from pT1 (164.423±108.170) to pT2 (195.000±83.367) and pT3 (266.667±57.735), as well as from grade 1 (75.000±98.742) to grade 2 (201.250±93.695) (*P*<0.05). However, in grade 3, the staining score decreased slightly (173.333±110.151). The chromophobe histological subtype presented with a higher staining score (275.000±35.355) than the clear cell type (171.724±107.614) or papillary type (173.333±75.056), but the difference was not significant.

**Figure 2:**
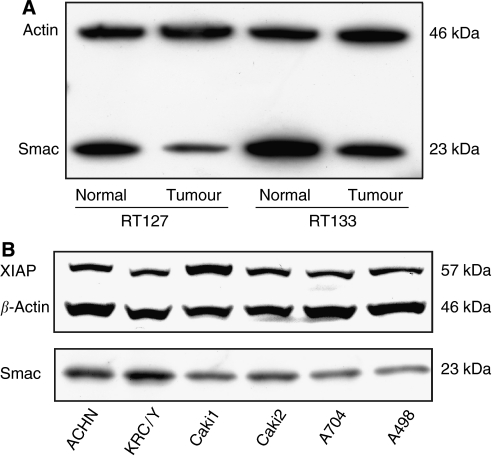
Western blot analysis of XIAP and Smac expression in paired tissue samples from normal kidney and RCC (**A**). Smac levels were lower in RCC than in normal kidney. Expression of XIAP and Smac in a panel of RCC cell lines (**B**). Both molecules were ubiquitously expressed, with the highest levels of XIAP in Caki1 cells. *β*-Actin was used as a control for loading.

The presence of both proteins was confirmed by western blotting of selected pairs of normal–tumour tissue samples (Figure 2A; data not shown) and Smac levels were lower in tumour tissues than in paired normal kidneys. XIAP protein was detected in all RCC cell lines examined and the expression differed slightly among the cells, with the highest level in Caki1 cells (Figure 2B). A Smac-specific band was also detected in all cell lines, with the highest level of expression in KRC/Y and the lowest in A498 (Figure 2B).

### XIAP shRNA stable clones

**Figure 3:**
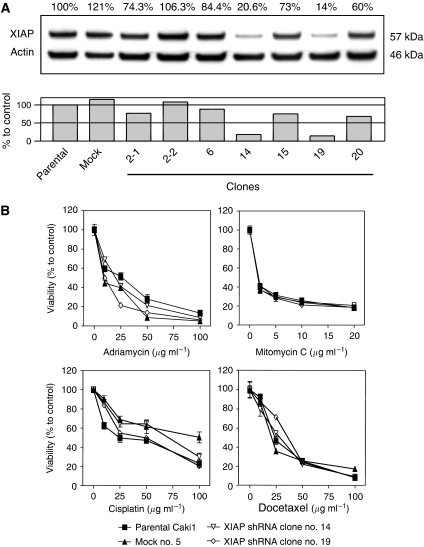
Western blot analysis of XIAP in Caki1 cells transfected with an XIAP-targeting shRNA vector (**A**). Figures above the panel indicate normalised expression (%). Clone nos. 14 and 19 with the lowest levels of XIAP were selected for further experiments. The relative viability (MTS assay) of Caki1 parental cells, mock-transfected cells, and clone nos. 14 and 19 treated with the indicated doses of adriamycin, mitomycin C, cisplatin, and docetaxel for 24 h is shown (**B**).

**Figure 4:**
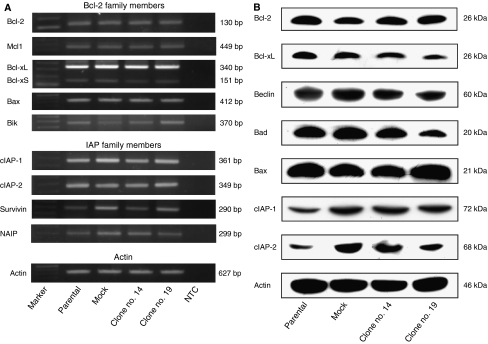
RT–PCR analysis (**A**) and western blot analysis (**B**) for Bcl-2 and IAP family members in the Caki1 parental cells, mock-transfected cells, and clone nos. 14 and 19. *β*-Actin was used as a control for loading. The messengers of all examined genes were present in the cells and remained unchanged. Apoptosis-related proteins were also not changed except cIAP-1 and cIAP-2, which were slightly increased in mock transfectants and clone nos. 14 and 19, which presumably reflects the effect of cellular stress during transfection and selection process.

**Figure 5:**
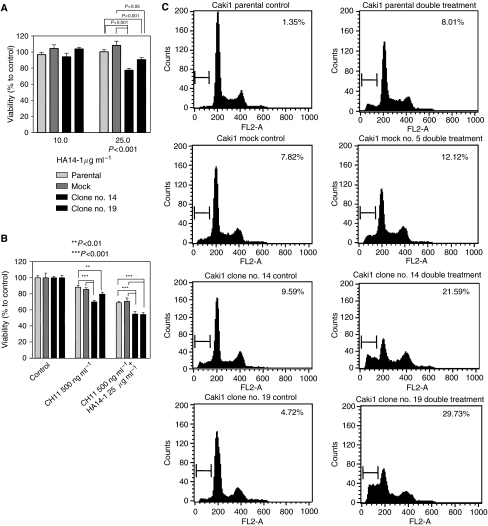

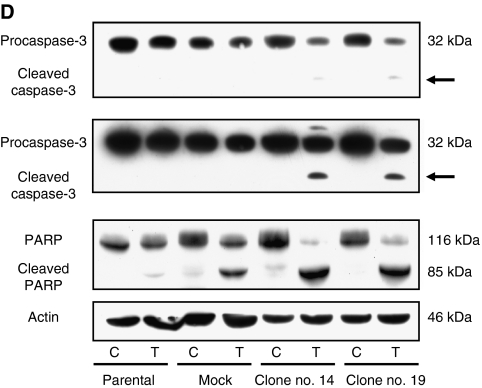
Relative viability (MTS assay) of Caki1 parental cells, mock-transfected cells, and clone nos. 14 and 19 treated with the small-molecule Bcl-2 inhibitor HA14-1 for 24 h (**A**). The same cells treated with either CH11 (500 ng ml^−1^) Fas-stimulating antibody or a combination of CH11 (500 ng ml^−1^) and HA14-1 (25 *μ*g ml^−1^) for 24 h (**B**). One-way ANOVA with a Tukey post-test to compare all pairs of values was used. (**C**) Cells untreated (left column) or treated with a combination of CH11 (500 ng ml^−1^) and HA14-1 (25 *μ*g ml^−1^) for 24 h (right column) were fixed and stained with PI and further analysed on a flow cytometer to detect sub-G1 population – a late apoptotic event. Figures indicate percentage of cells of sub-G1 population. A drastic increase in sub-G1 population was observed in clone nos. 14 and 19. (**D**) Western blot of untreated control cells (C) or cells treated with a combination of CH11 (500 ng ml^−1^) and HA14-1 (25 *μ*g ml^−1^) for 24 h (T). Membranes were probed with anti-caspase 3 or anti-PARP antibody. The upper panel presents caspase-3 blot after normal exposure showing a decrease in zymogen caspase 3 in clone nos. 14 and 19, and the lower panel shows overexposed blot with clearly observed caspase-3-cleaved fragments in clone nos. 14 and 19 after double treatment. PARP cleavage was also observed, indicating activation of biochemical apoptotic pathways. *β*-Actin was used as a control for loading.

To explore the effect of a constitutional decrease in XIAP on cell survival and susceptibility to apoptosis, we generated stable Caki1 clones of shRNA expressing vector targeting XIAP (TA0025-4) as well as a control vector as described in Materials and Methods. In the two clones selected for further experiments, XIAP protein was suppressed by 79.4% in clone no. 14 and by 86% in clone no. 19 (Figure 3A). The stable transfectants and mock clone did not demonstrate any morphological difference from the parental cells. We studied sensitivity of the cells to conventional anticancer drugs, which are considered to exert their effect through induction of apoptosis and are widely used in urology to treat bladder, testicular, and prostate cancer. Unexpectedly, all the cells, including parental cells and mock clones, showed equal sensitivity to adriamycin, mitomycin C, cisplatin, and docetaxel (Figure 3B). As there is redundancy in the IAP family and other members can be reciprocally upregulated to substitute for XIAP, we performed RT–PCR and western blotting for apoptosis-related genes. The messengers of Bcl-2 and IAP family members examined were present and did not show drastic changes among the parental cells, mock transfectants, and clone nos. 14 and 19 (Figure 4A). Furthermore, to check for protein levels, we performed western blot analysis. The levels of Bcl-2 family members were unchanged but those of c-IAP1 and c-IAP2 (Figure 4B) increased slightly in mock cells and transfectants. This was not a specific modulation due to downregulation of XIAP, as it was also observed in mock cells. Rather, it can be explained by the effect of cellular stress due to transfection and selection process. The knockdown of XIAP by RNAi sensitised the cells to HA14-1, a small-molecule Bcl-2 inhibitor (Figure 5A), but this effect was moderate and observed only at a single concentration of HA14-1 (25 *μ*g ml^−1^). At this dose, the overall *P*-value was less than 0.0001. *P*-values between each pair of the cells calculated with the post-test are presented in Figure 5A. As XIAP inhibition sensitised chronic lymphocytic leukaemia cells (Kater *et al*, 2005) and RCC cells (Mizutani *et al*, 2007) to Fas-induced apoptosis, we treated the parental cells, mock transfectants, and clone nos. 14 and 19 with CH11 IgM Fas-stimulating monoclonal antibody (Figure 5B). Overall, the viability of clone nos. 14 and 19 was significantly lower than that of the parental cells or mock transfectants (*P*<0.0001). *P*-values between each pair of the cells calculated with the post-tests are presented in Figure 5B. Double treatment with CH11 and HA14-1 resulted in a significant (*P*<0.0001) decrease in the viability of clone nos. 14 and 19 (Figure 5B), and a synergistic effect of the two compounds was observed in parental cells, mock transfectants, and clone no. 19. As the decrease in relative viability can be attributed to decreased cell proliferation, nonspecific toxicity, and non-apoptotic cell death, we performed additional experiments to clear this point. There was no difference in BrdU incorporation, which is a marker of cell proliferation, between untreated and treated cells (data not shown). The proportion of annexin V-positive cells, which is an early apoptosis marker, increased in double-treated cells from 5.0% (parental) and 3.05% (mock no. 5) to 6.03% (clone no. 14) and 9.41% (clone no. 19). Furthermore, there was a drastic increase in sub-G1 population after PI staining in clone nos. 14 (21.59%) and 19 (29.73%) compared to parental (8.01%) and mock transfectants (12.12%) (Figure 5C). This was accompanied with PARP and caspase-3 cleavage (Figure 5D, the upper panel presents caspase-3 blot after normal exposure demonstrating consumption of the proform of the protein and the lower panel shows overexposed blot with clearly observed caspase-3-cleaved fragments in clone nos. 14 and 19 after double treatment), indicating activation of biochemical apoptotic pathways. These data confirm the hypothesis that the decrease in viability of clone nos. 14 and 19 was due to acceleration of apoptosis.

### Smac-mimicking peptide

**Figure 6:**
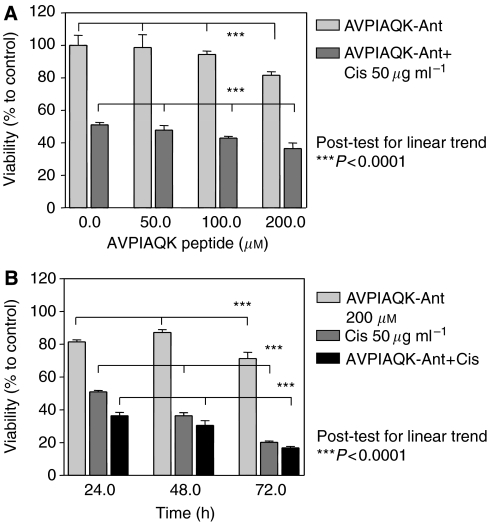
Relative viability (MTS assay) of Caki1 cells treated with indicated concentrations of Smac-Ant peptide (AVPIAQK) alone or in combination with cisplatin (50 *μ*g ml^−1^) for 24 h (**A**). The cells were exposed to 200 *μ*M Smac-Ant peptide (AVPIAQK), cisplatin (50 *μ*g ml^−1^), or a combination of the two agents for 24, 48, and 72 h (**B**). One-way ANOVA with post-test for linear trend was used to analyse the data.

**Figure 7:**
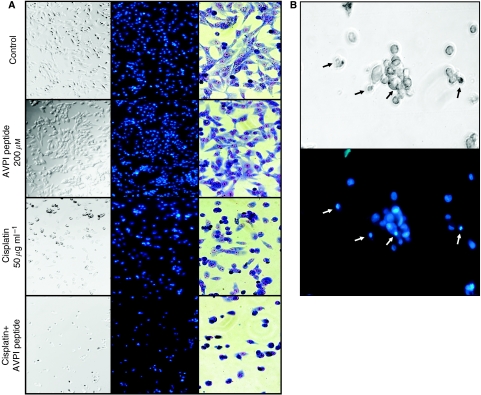
Phase contrast (left), Hoechst 33342 (middle), and Giemsa (right) photographs of Caki1 cells treated with 200 *μ*M Smac-Ant peptide, 50 *μ*g ml^−1^ cisplatin, or a combination of the two agents for 24 h (**A**). (**B**) Higher magnification of phase contrast (upper panel) and Hoechst 33342 (lower panel) photographs of the same field of Caki1 cells treated with a combination of Smac-Ant peptide (AVPIAQK) (200 *μ*M) and cisplatin (50 *μ*g ml^−1^). Cells undergo typical apoptotic morphological changes (condensed fragmented nuclei with formation of apoptotic bodies) as indicated by arrows.

Transfection of the ACHN and Caki1 cells with Smac-N7 or control peptide using either Chariot or BioPORTER protein transfection reagent resulted in pronounced nonspecific cytotoxicity. We switched from unbound peptide to antenopedia-linked peptide, which can be delivered to the cells without additional transfection reagent. Treatment of Caki1 cells with various doses of the antenopedia-bound Smac-mimicking peptide resulted in a significant dose-dependent decrease in viability (overall *P*=0.0031; linear trend *P*<0.0001, *r*^2^=0.804) (Figure 6A) and a further effect in combination with cisplatin was observed (overall *P*=0.0027; linear trend *P*<0.0001, *r*^2^=0.8419) (Figure 6A). The time course demonstrated increased toxicity with time in the case of the peptide alone and also in combination with cisplatin (linear trend *P*<0.0001, *r*^2^=0.2892 and *P*<0.0001, *r*^2^=0.842, respectively) (Figure 6B). Addition of the Smac-mimicking peptide to the medium containing cisplatin accelerated apoptosis, as typical morphological changes were observed (Figure 7A and B).

## DISCUSSION

The present immunohistochemical data are consistent with previous reports that XIAP expression was increased in RCC compared with normal kidney and XIAP levels (Ramp *et al*, 2004; Yan *et al*, 2004; Mizutani *et al*, 2007) correlated with tumour progression. Despite several previous attempts, there is still no consensus on the significance of Smac to the malignant potential of RCC (Yan *et al*, 2004; Mizutani *et al*, 2005). Here, we found that Smac levels were lower in RCC than in normal kidney; however, the levels were similar in various tumour tissues. In our limited series, the distribution is skewed in favour of low-stage (26 (76.5%) were stage 1) and low-grade (90% were grade 1–2) tumours.

Previously, we demonstrated the importance of XIAP to the malignant potential of transitional cell cancer (TCC) and the chemosensitisation of TCC by XIAP antisense oligonucleotides (Bilim *et al*, 2003). A decrease in the levels of XIAP has been reported to sensitise cancer cells to cytotoxic T lymphocytes (Kashkar *et al*, 2006), radiation (Ohnishi *et al*, 2006), and anticancer drugs (Hu *et al*, 2003). XIAP antisense oligonucleotides potentiated Fas/TRAIL-induced apoptosis in RCC (Mizutani *et al*, 2007). To our knowledge, RNAi technology has not been used previously to downregulate the expression of XIAP in RCC cells and there are no reports on the functional significance of XIAP in RCC. Here, XIAP downregulation did not sensitise RCC cells to cisplatin, docetaxel, adriamycin, or mitomycin C. There are several putative explanations for this phenomenon. One is the absence of caspases. However, as demonstrated previously, caspase expression was preserved in RCC from patients and cell lines (Gerhard *et al*, 2003), and caspase 3 was detected in Caki1 cells (Figure 5D). On the other hand, the redundancy of IAP family members or the importance of high Bcl-2 levels cannot be ruled out.

Renal cell carcinoma expresses high levels of Bcl-2 expression without gene amplification, as demonstrated previously by us (Tomita *et al*, 1996a). This fact can explain RCC's high resistance to conventional chemo- and radiotherapy, and the absence of Bcl-2 had a positive predictive value for the response to immunotherapy in metastatic RCC, as was disclosed in our recent study (Maruyama *et al*, 2006). Proapoptotic Bcl-2 family members Bid, Bak, and Bax are essential for the release of cytochrome *c* and Smac from mitochondria (Kandasamy *et al*, 2003), whereas Bcl-2 and Bcl-xL prevent mitochondrial release of Smac (Sun *et al*, 2002). The downregulation of Bcl-2 expression by antisense PODN resulted in Smac's translocation to the cytoplasm in T24 UC cells (our own unpublished observation). High levels of Bcl-2 can explain the inability of downregulation of XIAP expression to sensitise RCC cells to chemotherapeutic drugs, which are believed to trigger an intrinsic, mitochondria-mediated apoptotic pathway. It is feasible that proapoptotic signalling is blocked by Bcl-2 at the level of mitochondria, upstream of XIAP. In this light, the sensitisation to Fas-induced apoptosis caused by the knockdown of XIAP (Figure 5B) is easy to understand, as ligands to TNF family receptors can directly activate downstream caspases bypassing mitochondria. We tried targeting Bcl-2 with a small-molecule inhibitor that induced cell death more efficiently (*P*<0.0001; Figure 5A) in XIAP-knockdown cells compared to parental cells or mock transfectants. The engagement of Fas receptors with simultaneous targeting of Bcl-2 resulted in a significant (*P*<0.0001) decrease in viability of clone nos. 14 and 19 (Figure 5B) and a synergistic effect was observed in parental cells, mock transfectants, and clone no. 19.

The decrease in cellular viability due to the combination of CH11 and small-molecule Bcl-2 inhibitor was a result of enhanced apoptosis. The presence of annexin V-positive cells and the drastic increase in sub-G1 population (Figure 5C) confirm this statement. Apoptosis was easily induced in clone nos. 14 and 19, compared with parental and mock cells. This is in agreement with the results of viability assay. The cleaved product of caspase 3 was observed only in clone nos. 14 and 19, but not in parental or mock transfectants, and PARP cleavage was most prominent in the cells with XIAP knockdown (Figure 5D). Thus, combined targeting of XIAP with Bcl-2, which blocks apoptotic signalling at the mitochondrial level and downstream of it, with simultaneous stimulation of Fas facilitates apoptosis in RCC.

Redundancy of IAP family members is another possibility. It is known that Smac peptide acts not only on XIAP but also on other IAP family members: melanoma IAP, survivin, cIAP1, and cIAP2, the last three being expressed in RCC and their expression confirmed in Caki1 cells (Figure 4A). Various approaches are being used to develop Smac-mimicking reagents for inhibiting XIAP. One approach is to design small molecules. Another is to use N-terminal Smac peptides. Smac peptide enhanced the apoptosis induced by chemotherapeutic drugs (Arnt *et al*, 2002; Yang *et al*, 2003b) or TRAIL (Guo *et al*, 2002). Thus, Smac-N7 peptide efficiently sensitised the cells to cisplatin (Figures 6, 7), which was not achieved by only targeting XIAP.

Consequently, to overcome the resistance of RCC cells to apoptosis, a combination of treatments targeting several molecules is necessary. The engagement of a TNF receptor-mediated pathway simultaneously with the downregulation of XIAP expression with or without the targeting of Bcl-2 is promising. Alternatively, targeting several IAP family members with Smac-N7 peptide in combination with conventional chemotherapy can be done. The main problem with applying these approaches to the clinical setting is the absence of a suitable drug delivery system for nucleic acids (siRNAs or antisense oligonucleotides) and peptides. In this light, the creation of small molecular targeting drugs seems to be more appropriate, with the application of RNAi and peptides to search for promising molecular targets.
